# Developmental Mechanism of Limb Field Specification along the Anterior–Posterior Axis during Vertebrate Evolution

**DOI:** 10.3390/jdb4020018

**Published:** 2016-05-19

**Authors:** Mikiko Tanaka

**Affiliations:** Graduate School of Bioscience and Biotechnology, Tokyo Institute of Technology, B-17, 4259 Nagatsuta-cho, Midori-ku, Yokohama 226-8501, Japan; mitanaka@bio.titech.ac.jp; Tel.: +81-45-924-5722

**Keywords:** *Hox*, retinoic acid, *Tbx4*, *Tbx5*, limb field specification, evolution

## Abstract

In gnathostomes, limb buds arise from the lateral plate mesoderm at discrete positions along the body axis. Specification of these limb-forming fields can be subdivided into several steps. The lateral plate mesoderm is regionalized into the anterior lateral plate mesoderm (ALPM; cardiac mesoderm) and the posterior lateral plate mesoderm (PLPM). Subsequently, *Hox* genes appear in a nested fashion in the PLPM and provide positional information along the body axis. The lateral plate mesoderm then splits into the somatic and splanchnic layers. In the somatic layer of the PLPM, the expression of limb initiation genes appears in the limb-forming region, leading to limb bud initiation. Furthermore, past and current work in limbless amphioxus and lampreys suggests that evolutionary changes in developmental programs occurred during the acquisition of paired fins during vertebrate evolution. This review presents these recent advances and discusses the mechanisms of limb field specification during development and evolution, with a focus on the role of *Hox* genes in this process.

## 1. Introduction

**Figure 1:**
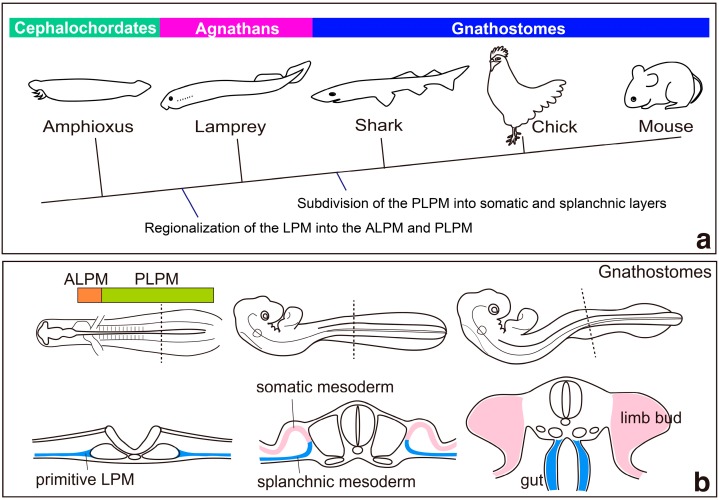
Model for the evolution of the lateral plate mesoderm (LPM); (**a**) a phylogenetic tree indicating the probable timing of the regionalization and subdivision of the LPM. See text for details. Modified after [4]. (**b**) development of the LPM in chick embryos. Bottom panels show schematic cross-sections at the wing level. The LPM is regionalized into the anterior lateral plate mesoderm (ALPM) and the posterior lateral plate mesoderm (PLPM). Then, the developing LPM splits into somatic and splanchnic mesoderm. Subsequently, somatic mesodermal cells proliferate and form limb buds, whereas splanchnic mesodermal cells contribute to gut formation. Modified after [9].

Paleontological evidence suggests that acquisition of the first pairs of fin-like structures occurred in the lineage of ancestral agnathans (jawless vertebrates; Figure 1a) [1,2]. The cephalochordate amphioxus and the agnathan lampreys are thought to have diverged prior to the acquisition of paired fins (Figure 1a) [3], and thus these animals could be good models for developmental comparisons to gain insights into the evolutionary sequence that lead to the acquisition of the limb-forming fields in the lateral plate mesoderm.

In gnathostomes (jawed vertebrates), limb buds appear as small bulges protruding from the body trunk at a specific position along the body axis during the development of gnathostome embryos. Specification of limb fields, which is tightly correlated with the initiation of limb bud formation, involves multiple steps [4]. The lateral plate mesoderm is regionalized into the anterior lateral plate mesoderm (ALPM; cardiac mesoderm) and the posterior lateral plate mesoderm (PLPM), which includes the presumptive limb-forming fields [5,6]. Retinoic acid signaling seems to play pivotal roles in this process [5,6]. In the PLPM, *Hox* genes are expressed in a nested fashion along the anterior–posterior axis and appear to be involved in the regionalization of the PLPM into forelimb, interlimb flank and hindlimb fields [7,8]. Then, the lateral plate mesoderm thickens and splits into the somatic and splanchnic layers sequentially from the anterior to posterior region of the embryos (Figure 1b) [9]. Subsequently, expression of limb initiation genes appears in the presumptive limb-forming fields [10]. Initiation of limb buds needs to occur at a precise position along the body axis, although little has been known until recently about the molecular mechanism linking limb positioning and initiation. Analyses of chick and mouse embryos, however, revealed that the *Hox* proteins that define the axial position of the limb-forming fields directly activate the transcription of the forelimb initiation gene, *Tbx5* [11,12]. Furthermore, developmental analyses of limbless amphioxus and lampreys have provided evidence for the sequential events that occurred during the acquisition of limb-forming fields in the body of vertebrates during evolution [4]. This review highlights these recent advances and discusses the mechanisms of limb field specification during development and during vertebrate evolution, with a special focus on the function of *Hox* genes.

## 2. Regionalization of the Lateral Plate Mesoderm into the ALPM and the PLPM: Retinoic Acid and *Hox* Genes

During the development of gnathostome embryos, the lateral plate mesoderm is regionalized into the ALPM and the PLPM, including the presumptive limb-forming fields [5,6]. Recent developmental studies on zebrafish and mouse embryos suggested that such a regionalization of the lateral plate mesoderm is necessary to provide an environment permissive for forelimb-forming fields and that retinoic acid signaling has pivotal roles in this process [5,6]. In zebrafish, mutants for *retinaldehyde dehydrogenase 2* (*raldh2*) lack pectoral fin buds (forelimb buds) [13,14], and embryos treated with retinoic acid inhibitor show the posterior expansion of the heart field and fail to initiate formation of pectoral fin buds [5]. During this process, retinoic acid signaling induces the expression of *hoxb5b* within the forelimb fields [5]. As *hoxb5b* restricts the number of atrial cells, it also restricts the posterior extension of the heart field to determine the anterior boundary of forelimb-forming fields, although its function is dispensable for forelimb formation [5]. Chick and mouse *Hoxb5* orthologs have been implicated as direct targets of retinoic acid signaling [15,16,17]. Moreover, in mouse *Hoxb5* mutants, the shoulder girdles shift their position, although morphological abnormalities of the heart have not been reported [18]. Thus, it is suggested that *Hoxb5* mutants might have subtle cardiac defects, or be lethal, or other mouse *Hox* genes might have roles similar to those of zebrafish *hoxb5b* [5]. Multiple *Hox* genes are expressed in the heart fields of mouse and chick embryos [19,20]. However, heart phenotypes associated with the genetic inactivation of *Hox* genes have rarely been observed, which is probably due to the functional redundancy of paralogous *Hox* genes [21]. One notable exception is mouse *Hoxa3* null mutants, which exhibit heart defects, including hypertrophy of the atria and the enlargement of the chief veins [22]. Heart phenotypes seen in *Hoxa3* mutants, thus, have been implicated as a possible outcome of the expansion of the heart field [5].

Numerous studies on retinoic acid signaling in relation to the anterior-posterior patterning of the heart suggested roles for *Hox* genes in this process [21,23]. Treatment with retinoic acid enhances the expression of *Hoxa4, Hoxd3 and Hoxb5* in chicken cardiogenic tissue [20]. In addition, *Hoxb1, Hoxa1* and *Hoxa3*, downstream targets of retinoic acid signaling, define the distinct progenitor sub-domains of the secondary heart field in mouse embryos [24]. Furthermore, retinoic acid signaling is correlated with limb initiation and positioning in zebrafish, chick and mouse embryos [5,6,25,26]. Chick embryos treated with disulfiram, an inhibitor of retinoic acid synthesis, show hypoplasia, or a positional shift of the forelimb bud [25]. In mouse *Raldh2* mutants, heart-forming fields expanded posteriorly, and forelimb initiation fails [6], as seen in zebrafish *raldh2* mutants [5]. Thus, retinoic acid signaling seems to have a role in the regionalization of the lateral plate mesoderm into the ALPM and the PLPM [6,26,27]. In addition, in the pharyngeal endoderm and mesoderm of *Raldh2* mouse mutants, *Hoxa1* and *Hoxb1* transcript levels are reduced [27], suggesting that the role of retinoic acid signaling in regionalizing the lateral plate mesoderm via regulation of *Hox* genes is conserved in mouse embryos.

An alternative model for the function of retinoic acid signaling in the regionalization process of the lateral plate mesoderm along the anterior–posterior axis has been proposed by Duester’s group. They suggested that retinoic acid signaling delimits the cardiac and epiblast *Fgf8*-positive domains, and provides a permissive environment for the induction of forelimb (but not hindlimb) development [6]. Chromatin immunoprecipitation analysis revealed that all three retinoic acid receptor (RAR) isoforms indeed bind to the retinoic acid responsive elements near the *Fgf8* promoter, suggesting that retinoic acid signaling directly represses *Fgf8* expression [28]. Interestingly, transgenic zebrafish embryos expressing ectopic Fibroblast growth factor (Fgf) signaling show expansion of the heart field and failure of initiation of pectoral fin development [29]. These results seem to support the view that FGF signaling is involved in the regionalization of the lateral plate mesoderm and provides a permissive environment for forelimb induction [6].

**Figure 2:**
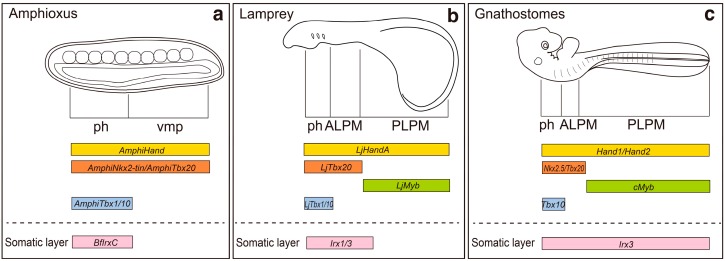
Schematic model for regionalization and differentiation of the ventral mesoderm and the lateral plate mesoderm (LPM) in (**a**) amphioxus, (**b**) lampreys and (**c**) gnathostomes. Yellow, orange, green and blue bars represent the distribution of molecular markers for the ventral mesoderm/LPM (*AmphiHand*, *LjHandA*, *Hand1*, *Hand2*), the pharyngeal mesoderm (ph) and ALPM (*AmphiNkx2-tin, AmphiTbx20, LjTbx20, Nkx2.5, Tbx20*), the PLPM (*LjMyb, cMyb*), and the pharyngeal mesoderm (*AmphiTbx1/10, LjTbx1/10, Tbx10*), respectively. Pink bars represent the distribution of *Irx3* orthologs (*BfIrxC, Irx1/3, Irx3*), which are expressed in the somatic layers of the LPM. vmp, ventral mesoderm posterior to the pharynx. See text for gene expression references. Modified after [4,38]

During embryonic development, regionalization of the lateral plate mesoderm into the ALPM and the PLPM provides a permissive environment for forelimb induction. Therefore, regionalization of the lateral plate mesoderm might have been a crucial step for acquisition of the limb-forming field during the evolution of vertebrates. In vertebrates, the heart is located at the anterior end of the body trunk, whereas the primitive heart of amphioxus—consisting of peristaltic blood vessels—is located at the posterior-ventral part of the body [30]. Recently, the distribution of the ALPM and the PLPM has been examined in amphioxus embryos by using molecular markers (Figure 2) [4]. In amphioxus, *AmphiHand*, an ortholog of *Hand1* and *Hand2*, which are expressed throughout the lateral plate mesoderm of zebrafish, chick and mouse embryos [31,32,33], is expressed throughout the ventral mesoderm (Figure 2) [4]. Interestingly, in amphioxus, both *AmphiNkx2-tin* and *AmphiTbx20*—orthologues of *Nkx2.5* and *Tbx20*, which are expressed in the cardiac mesoderm [32,34,35,36]—are also expressed throughout the ventral mesoderm. These results suggest that the amphioxus ventral mesoderm is not molecularly regionalized into cardiac *versus* posterior ventral mesoderm (Figure 2) [4]. In the limbless agnathan lamprey, *Lethenteron japonicum Tbx20* (*LjTbx20*) is expressed in the anterior part of the lateral plate mesoderm [37], whereas *LjMyb*, an orthologue of *c-myb* that is expressed in the PLPM, is expressed in mesodermal cells posterior to the *LjTbx20*-positive area (Figure 2) [4]. These results suggest that the lateral plate mesoderm of lamprey, a limbless agnathan, is regionalized into the ALPM and the PLPM as seen in gnathostomes (Figure 2) [4].

In lampreys, expression of *LjTbx1/10*, an ortholog of *Tbx1* and *Tbx10* that is a marker of pharyngeal mesoderm [39], is restricted to the pharyngeal mesoderm [40]. Importantly, expression of *AmphiTbx1/10* is also restricted to the pharyngeal ventral mesoderm [41], suggesting that the ventral mesoderm of amphioxus is indeed regionalized into the pharyngeal ventral mesoderm and ventral mesoderm caudal to the pharynx (Figure 2) [4]. Therefore, it seems that the system to regionalize the ventral mesoderm along the anterior–posterior axis was already established in the cephalochordate lineage, although the ventral mesoderm was not yet separated into the cardiac and posterior ventral mesoderm [4].

**Figure 3:**
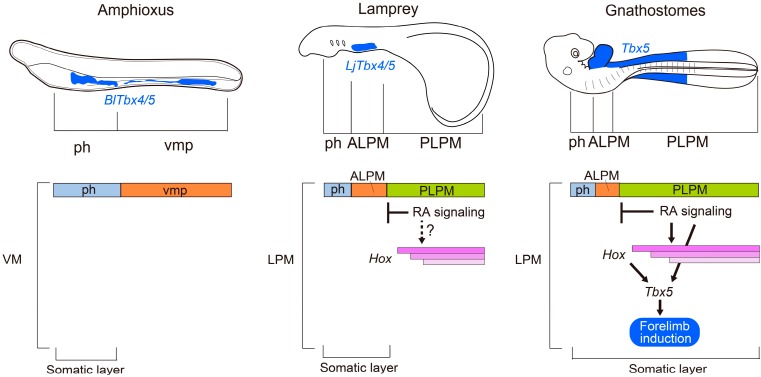
Schematic model for the acquisition of forelimb-forming fields during evolution. Light blue, orange and green bars represent the pharyngeal mesoderm (ph), ventral mesoderm posterior to the pharynx (vmp)/ALPM and PLPM, respectively (see Figure 2 for details). The ventral mesoderm (VM)/LPM is regionalized into the ALPM and PLPM in lampreys and gnathostomes, but not in amphioxus, and retinoic acid signaling seems to be involved in this process. Nested expression of *Hox* genes (pink bars) is observed in lampreys and gnathostomes, but not in amphioxus. Blue regions in the embryos are expression domains of *Tbx5* orthologs (*BlTbx4/5*, *LjTbx4/5*, *Tbx5*) in the VM/LPM. In amphioxus, *BlTbx4*/*5* (blue) appears in both ph and vmp, similar to cardiac markers (*AmphiNkx2.5-tin/AmphiTbx20*; see Figure 2). In lampreys, *LjTbx4/5* expression (blue) is expressed in the ALPM; it is not expressed in the unseparated PLPM. In gnathostomes, retinoic acid signaling, as well as Hox proteins, directly activates expression of *Tbx5* (blue) in the somatic layer of the PLPM to induce forelimb formation [11,49,50]. See text for additional references and details.

Previous studies suggest that retinoic acid signaling is involved in the patterning of various organs along the anterior–posterior axis both in amphioxus and lampreys [42,43,44,45,46,47]. RARs have been identified in amphioxus [42] and lampreys [43]. Furthermore, treatments with excess retinoic acid or retinoic acid inhibitors affect the anterior–posterior patterning of amphioxus and lampreys [42,43,44,45,46,47]. Interestingly, lamprey embryos treated with retinoic acid fail to form a heart [47], supporting the view that retinoic acid has retained its importance for the regionalization of the lateral plate mesoderm into the ALPM and the PLPM during evolution (Figure 3) [4]. Therefore, it is likely that retinoic acid signaling and its roles in the anterior–posterior patterning were present in the common ancestor of vertebrates and amphioxus. However, a recent study on amphioxus embryos revealed that the function of retinoic acid signaling in anterior–posterior patterning of somites in amphioxus might not be the same as in vertebrates [48]. Bertrand *et al.* showed that retinoic acid signaling has critical roles in somitogenesis in amphioxus; however, it does not antagonize Fgf signaling. Therefore, the anterior–posterior patterning system controlled by mutual inhibition between retinoic acid and Fgfs during somitogenesis might have been acquired after the divergence of amphioxus and ancestral vertebrates [48]. Zhao *et al.* proposed that, during mouse embryogenesis, antagonism between retinoic acid and *Fgf8* provides the permissive environment for the forelimb-forming field in the PLPM [6]. Thus, we should consider the possibility that alteration of the distribution of retinoic acid in the lateral plate mesoderm and/or acquisition of a novel antagonistic function of retinoic acids and Fgfs might have been critical for subdividing the lateral plate mesoderm into the ALPM and the PLPM during vertebrate evolution (Figure 3).

In gnathostomes, several lines of evidence indicate that retinoic acid signaling regulates the transcription of *Hox* genes and leads to the regionalization of the lateral plate mesoderm along the anterior–posterior axis [5,21,23,27]. However, transcripts of *Hox* genes have not yet been observed in the segmented mesoderm (the progenitor of the ventral mesoderm) of amphioxus (Figure 3) [51,52]. In amphioxus, retinoic acid signaling regulates the expression of *Hox* genes in anterior–posterior patterning of the endoderm, central nervous system and ectoderm at the neurula stage [45,53,54]. In addition, retinoic acid signaling directly regulates expression of *Hox* genes to pattern the anterior–posterior axis during gastrulation [55], suggesting that the regulatory mechanism of *Hox* gene transcription by retinoic acid signaling has already been established in the common ancestors of amphioxus and ancestral vertebrates. As the nested expression of *Hox* genes has been observed in the PLPM of both lamprey and gnathostome embryos [4,7], the acquisition of the novel expression domain for *Hox* genes in the lateral plate mesoderm—possibly via alteration of distribution of retinoic acid and/or the acquisition of a novel function of retinoic acid signaling—seems to be required for the regionalization of the lateral plate mesoderm into the ALPM and the PLPM (Figure 3).

In lamprey embryos, *Hox* genes are indeed expressed in a nested fashion in the PLPM [4], although limb formation is not initiated in this region (Figure 3). In gnathostomes, subsequent to the regionalization of the lateral plate mesoderm into the ALPM and the PLPM, the lateral plate mesodermal cells proliferate and are subdivided into the somatic and splanchnic layers beginning from the anterior toward the posterior part of the body (Figure 1b) [9]. Limb buds then appear as protrusions of the somatic layers in the PLPM. In contrast, in lampreys, the lateral plate mesoderm is subdivided into the somatic and splanchnic layers in the ALPM, but not in the PLPM (Figure 2) [4]. Thus, it is possible that lampreys cannot form limb buds because the underdeveloped PLPM does not split into the somatic and splanchnic layers [4]. Until recently, subdivision of the PLPM had not been observed during embryogenesis of any teleost fishes. However, a recent histological analysis of pufferfish (*Takifugu* *niphobles*) embryos at a very early stage (eight-somite stage) revealed that the lateral plate mesoderm is indeed subdivided into somatic and splanchnic layers both in the ALPM and the PLPM [56]. Importantly, cells from the PLPM of lampreys contribute to the formation of peritoneal epithelium and blood cells, but not the body wall [4,57]. Therefore, it is likely that the proliferation and differentiation of the PLPM, as well as formation of the coelom cavity, might have been a crucial step in acquiring paired appendages during the evolution of vertebrates [4,57]. In gnathostomes, *Forkhead box F* (*FoxF*) is expressed throughout the PLPM prior to the separation; subsequent to the subdivision, *FoxF* transcripts are restricted to the splanchnic mesoderm and *Iroquis homeobox 3* (*Irx3*) expression appears in the somatic mesoderm [9]. In lampreys, *LjIrx1*/*3* is expressed in the somatic layers of the ALPM, but not in the PLPM, although *LjFoxF* is expressed throughout the PLPM [4] (Figure 2). Furthermore, in amphioxus, *AmphiFoxF* is expressed from the pharynx to the posterior end of the body trunk [4], and *Branchiostoma floridae IrxC* (*BfIrxC*) is expressed in the ventral mesoderm at the level of the pharynx, but not in the ventral mesoderm posterior to the pharynx (Figure 2) [58]. Therefore, it is likely that the common ancestor of amphioxus and ancestral vertebrates had the *FoxF*-positive lateral plate mesoderm, and *Irx3*-positive somatic mesoderm seems to have appeared at the cardiac level after the divergence of ancestral chordates and vertebrates. The *FoxF*-positive unseparated PLPM seems to have been acquired prior to the lamprey lineages. The *Irx3*-positive somatic layers seem to have appeared in the PLPM after the divergence of lampreys and vertebrates [4], and provided permissive environment needed for emergence of fin-forming fields during evolution.

## 3. Specification of Limb-Forming Fields: *Tbx4*, *Tbx5* and *Hox* Genes

In gnathostomes, the lateral plate mesoderm is divided into the ALPM and the PLPM, and *Hox* genes seem to be involved in this process [5]. Although regionalization of the PLPM along the anterior–posterior axis by *Hox* genes has not been studied as extensively as in the nervous system or in somites, it has been proposed that expression patterns of *Hox* genes in the lateral plate mesoderm are somehow related to the specification of limb-forming fields [7]. Furthermore, the positioning of the forelimb buds at approximately the anterior border of the expression domains of *Hox5* and *Hox6* in the lateral plate mesoderm is conserved among zebrafish, chick and mouse [5,59,60,61]. Recently, it was shown that Hox proteins are involved in the regulation of the transcription of *T-box transcription factor 5* (*Tbx5*) (Figure 3) [11,12,49]. *Tbx5* is expressed in the anterior paired appendages of zebrafish, chick and mouse embryos [62,63,64] and initiates forelimb development [12,65,66,67,68,69]. In humans, mutations in *TBX5* cause Holt–Oram syndrome (OMIM 142900), an autosomal-dominant disorder characterized by abnormalities of the upper limbs and cardiac defects [70,71]. Zebrafish with mutations in *pbx4*, which encodes a Hox protein co-factor, fail to induce *tbx5* expression or initiate formation of pectoral fin buds [72]. In addition, developmental and molecular analyses of chick and mouse embryos revealed that rostral *Hox* genes expressed in the lateral plate mesoderm directly regulate the expression of *Tbx5* and thereby control the axial position of forelimb-forming fields (Figure 3) [11].

Recent analyses of mouse mutants showed that *Hox5* and *Hox9* genes are involved in establishing the anterior and posterior domains of the forelimb, respectively [73,74]. Loss of function of all three *Hox5* genes leads to anterior forelimb defects resulting from derepression of *Shh* expression [73]. In the anterior forelimbs, *Hox5* proteins interact with promyelocytic leukemia zinc finger (Plzf) and cooperatively mediate repression of *Shh* expression [73]. In contrast, an analysis of a *Hox9* quadruple knockout revealed that axial *Hox9* paralogs establish the posterior field of forelimbs by triggering the posteriorly restricted expression of *Hand2*, which directly activates *Shh* at the posterior margin [74].

In chick and zebrafish, Wnt/β-catenin signaling has key roles in the initiation of limb formation [67,75]. In chick embryos, *Wnt2b* is expressed in the intermediate mesoderm and the lateral plate mesoderm in the presumptive forelimb-forming region, whereas *Wnt8c* is expressed in the presumptive hindlimb-forming region [75], and the implantation of *Wnt2b*- or *Wnt8c*-expressing cells into the interlimb flank region leads to the formation of an additional limb via induction of ectopic *Fgf10* expression [75]. Consistent with this result, zebrafish embryos injected with *wnt2b* morpholinos show downregulation of *tbx*5 expression and fail to form pectoral fin buds [67]. In mouse embryos, however, Wnt ligands that act upstream of *Fgf10* in the limb-forming field have not yet been found. Furthermore, double knockout mice for both *Lef1* and *Tcf1*, which mediate Wnt signaling by forming a complex with β-catenin, form normal limb buds [76]. However, a recent study in mice showed that conditional removal of β*-catenin* from forelimb-forming regions leads to the reduction of *Tbx5* expression and incomplete forelimbs, suggesting that the function of β-catenin to regulate *Tbx5* transcription is conserved among zebrafish, chick and mouse [50]. The T-cell factor/lymphoid enhancer factor (TCF/LEF) binding sequence is required for the enhancer activity of the forelimb-specific *Tbx5* regulatory element, suggesting that β-catenin directly regulates *Tbx5* expression [50]. Furthermore, retinoic acid signaling directly activates transcription of *Tbx5* in the forelimb-forming fields (Figure 3) [50]. Taken together, retinoic acid signaling, β-catenin/LEF/TCF and anteriorly nested *Hox* proteins seem to cooperatively activate *Tbx5* transcription for inducing forelimb bud formation [50].

It has also been shown that caudally expressed *Hox* proteins, such as *Hoxc8*, *Hoxc9* and *Hoxc10*, can form a repressive complex and bind to the forelimb-specific regulatory sequences of *Tbx5* and repress its expression [49]. Interestingly, in chick embryos, transplantation of an Fgf-soaked bead into the interlimb flank region turns off *Hoxc9* expression when an ectopic wing bud is induced, whereas it turns on *Hoxc9* expression when an ectopic leg bud is induced [7]. Thus, these previous findings seem to support the view that *Hoxc9* acts as a repressor of *Tbx5* transcription [49]. In addition, rostrally expanded *Hoxc8* distribution is present in the body trunk of a limbless vertebrate, the python [77], supporting the view that *Hoxc8* represses *Tbx5* expression [49].

Molecular mechanisms that specify the hindlimb-forming fields are not as well characterized as those of the forelimb-forming fields. *Tbx4*, which is closely related to *Tbx5*, is specifically expressed in the presumptive hindlimb forming fields of chick, mouse, teleosts and catshark (*Scyliorhinus canicula*) [62,63,64,78,79], and its expression is suggested to be regulated by *Hox* genes [12] and *Pitx1* [12,80]. In addition, studies on transgenic mice, in which *Tbx5* is replaced by *Tbx4*, revealed that *Tbx4* is able to substitute for *Tbx5* function in the initiation of the forelimb bud [12], suggesting that Tbx4 has a similar role in the initiation of hindlimb formation. However, unlike *Tbx5* null mutants, mice deficient for *Tbx4* are able to initiate *Fgf10* expression in hindlimb fields and form truncated hindlimbs [81]. Additional studies in mice deficient for *Tbx4* revealed that *Tbx4* is crucial for initiating hindlimb outgrowth, but an additional factor(s) other than *Tbx4* is likely to be involved in this process [82]. *Islet1*, a LIM-homeodomain transcription factor that is specifically expressed in the hindlimb progenitors, was recently shown to be required for hindlimb initiation [83,84]. Early inactivation of *Islet1* as well as β-catenin in the lateral plate mesoderm leads to a failure to initiate hindlimb development [84]. Furthermore, *Islet1* is required for the nuclear accumulation of β-catenin and thereby for activation of the β-catenin pathway, and that the β-catenin pathway is required for the maintenance of *Islet1* expression [84]. *Islet1* seems to be a missing piece in the regulation of hindlimb-specific initiation [84].

*Pitx1*, which encodes a member of the paired-like family of homeodomain transcription factors, is expressed in the presumptive hindlimb-forming fields in chick, mouse, stickleback (*Gasterosteus aculeatus*) and catshark embryos [79,85,86,87]. In mice, *Pitx1* is required for normal initiation of hindlimb development by regulating transcription levels of *Tbx4* [85,88,89]. It has recently been suggested that in Liebenberg syndrome (OMIM 186550) [90], an autosomal-dominant malformation of the upper limbs, the upper limbs have undergone a partial homeotic hindlimb transformation, and that this phenotype is associated with genomic rearrangements at the *PITX1* locus [91].

In teleosts, the roles of *tbx4* and *pitx1* in pelvic fin formation seem to be more critical than their roles in the hindlimb formation of amniotes. In *tbx4* mutant zebrafish, expression of *fgf10* is not induced, and thus pelvic fin buds fail to initiate their formation at a very early stage of fin initiation [92]. In the spine-deficient morph of the three-spined sticklebacks, expressions of neither *pitx1* nor *tbx4* are detected in the lateral plate mesoderm at the level of the pelvic fin, and pelvic fin buds fail to initiate their formation [87]. Subsequently, the responsible regulatory sequence for pelvic spine deficiency was found within the pelvic fin-specific enhancer of *pitx1* in three-spined stickleback [93,94]. In teleosts, pelvic fin formation is significantly delayed as it is triggered by thyroid hormone at the time of the larva-juvenile transition [95] (for instance, three weeks post–fertilization in zebrafish). Thus, the levels of molecules that affect the limb initiation process in the pelvic fin-forming region of teleosts at the time of pelvic fin initiation are not identical to those in the presumptive hindlimb-forming region of early chick and mouse embryos. Although we cannot exclude the possibility that the functions of Pitx1 and/or *Tbx4* in the initiation of pelvic fin/hindlimb development in teleosts are different from those in mice, it is possible that the Pitx1-*Tbx4* pathway is sufficient to activate the initiation of pelvic fin outgrowth in teleost fishes.

A shift in limb position is also observed in chick and mouse embryos in which expression of *Gdf11* has been manipulated [96,97]. Mutant mice carrying a targeted deletion of *Gdf11* exhibit posterior displacement of the hindlimbs, and patterns of expression of *Hoxc6*, *Hoxc10* and *Hoxc11* in spinal cord and paraxial mesoderm also shift posteriorly [96]. In chick embryos, overexpression of *Gdf11* in the spinal cord leads to the rostral shift of both fore- and hindlimb [97]. In the spinal cord of chicks that overexpress *Gdf11*, expression of *Hoxc6*, *Hoxc8*, *Hoxc9* and *Hoxc10* shift rostrally and lead to the rostral shift of the lateral motor columns [97], which are defined by Hox6 and Hox10 [98,99,100]. Thus, the shift in limb buds in chick following the introduction of *Gdf11*-expressing constructs into the spinal cord was interpreted to be caused by a shift in the lateral motor columns [97]. *Gdf11* expression in the caudal part of the body is conserved in chick, mouse, zebrafish and Nile tilapia embryos [101,102,103]. Furthermore, in zebrafish injected with *gdf11* morpholinos, the pelvic fin shifts caudally, along with a posterior shift in *Hoxc10* expression in the spinal cord [103], suggesting that the Gdf11-controlled mechanism that positions the hindlimb along the body axis is likely to be conserved in gnathostomes [103,104]. Gdf11 signaling is a central modulator of the trunk-to-tail transition during mouse development [105]. In *Gdf11* mutants, the specification of the cloaca and the induction of the hindlimbs are significantly delayed, whereas precocious activation of Gdf11 signaling in the epiblast anteriorizes these structures with a concomitant reduction in trunk length [105]. In contrast, precocious activation of genes of the *Hox9*, *Hox10* and *Hox11* groups in the epiblast do not alter the phenotype, with one exception: transgenic mice expressing *Hoxb9* show only a slight anterior displacement of the hindlimbs by one somite [105]. Thus, Jurberg and his colleagues proposed that *Hox* genes do not play a major role in specifying the hindlimb position along the anterior–posterior axis during the trunk-to-tail transition [105]. In early stages of hindlimb induction, *Islet1* has a major role in establishing the posterior hindlimb field by regulating the Hand2-Shh pathway [84,106]. During the trunk-to-tail transition, Gdf11 directly activates *Islet1* to promote formation of the most posterior progenitors of the lateral plate mesoderm, such as the hindlimbs and cloacal tissue [105].

It has been suggested that the nested *Hox* gene expression in the lateral plate mesoderm is necessary for acquiring the first pair of appendages during the evolution of vertebrates [107]. In limbless amphioxus species *Branchiostoma floridae* and *Branchiostoma lanceolatum*, *Tbx4/5* is expressed throughout the ventral mesoderm (Figure 3) [108,109]. Interestingly, amphioxus *Tbx4/5* has the ability to initiate forelimb formation in mouse transgenic lines [110]. This apparent contradiction can be explained by the regionalization of the ventral mesoderm and expression of *Hox* genes mentioned above. In amphioxus, the ventral mesoderm is not molecularly regionalized into the cardiac and posterior ventral mesoderm [4], and *Hox* genes are not expressed in the ventral mesoderm progenitors [51,52], suggesting that *Tbx4/5* in the ventral mesoderm may contribute only to heart formation in amphioxus [4]. In lampreys, the lateral plate mesoderm is regionalized into the ALPM and the PLPM, and *LjHox5i* and *LjHox6w* are expressed in the PLPM in a nested fashion [4]. Nevertheless, lamprey *LjTbx4/5* transcripts are not present in the PLPM but are in the ALPM [4,37]. Although we do not know whether lamprey *Hox* genes have the conserved function of inducing *Tbx4/5* in the PLPM, one of the main reasons for limblessness seems to be the lack of separation of the PLPM into the somatic and splanchnic layers (Figure 3), which results in the contribution of cells from the PLPM to the peritoneal epithelium but not to the body wall [4,57].

## 4. Conclusions

**Figure 4:**
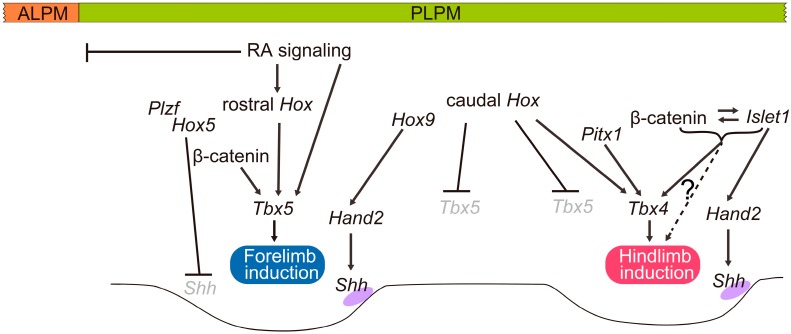
Genes implicated in the specification of the limb field during development of mouse embryos. Retinoic acid signaling is involved in the regionalization of the LPM into the ALPM and PLPM. In the PLPM, *Tbx5* and *Tbx4*, which are expressed in forelimb- and hindlimb-forming regions, respectively, play pivotal roles in limb initiation. Nested expression of *Hox* genes seems to be involved in the regionalization of the PLPM into forelimb, interlimb flank and hindlimb fields. In the forelimb level, rostrally expressed *Hox* proteins, retinoic acid signaling and β-catenin signaling cooperatively activate *Tbx5* transcription. Whereas caudally expressed *Hox* genes repress *Tbx5* to restrict its expression to the forelimb level, *Hox* genes and *Pitx1* have been suggested to activate *Tbx4* expression at the hindlimb level. *Islet1* is also required for hindlimb initiation. *Islet1* is required for the nuclear accumulation of β-catenin, and β-catenin is required for the maintenance of *Islet1* expression. In the forelimb posterior domain, *Hox9* genes are required to trigger *Hand2* expression in this region. In the forelimb anterior domain, *Hox5* genes and *Plzf* cooperatively mediate repression of *Shh* expression. In the hindlimb posterior domain, *Islet1* activates *Hand2* expression. It should be noted that the roles of *Hox* genes, RA signaling and β-catenin signaling are disputed. See text for references and details.

In gnathostomes, the lateral plate mesoderm is regionalized into the ALPM and PLPM, and *Hox* genes are expressed in a nested fashion along the anterior–posterior axis in the PLPM. Then, the lateral plate mesoderm splits into the somatic and splanchnic layers, and the expression of limb initiation genes appears in the limb-forming fields. Figure 4 summarizes the genes implicated in the specification of limb-forming fields in the PLPM during mouse development. *Tbx5* and *Tbx4* are expressed in the forelimb- and hindlimb-forming fields, respectively [63,64], and play crucial roles in the initiation of limb outgrowth [12,65,66,67,68,69]. In the forelimb-forming field, retinoic acid signaling and β-catenin signaling act cooperatively with rostrally expressed *Hox* genes to directly regulate *Tbx5* expression [11,50], whereas caudally expressed *Hox* genes repress *Tbx5* to restrict its expression to this field [49]. In the hindlimb-forming field, *Tbx4* expression, which is crucial for the induction of hindlimb development, is suggested to be activated by *Hox* genes [12] and *Pitx1* [12,85]. *Islet1-*mediated activation of β-catenin signaling is also required for hindlimb induction [84]. In the early forelimb field, *Hox9* paralogous genes are required for the expression of *Hand2* to establish the posterior domain [74], whereas *Hox5* genes and *Plzf* cooperatively mediate repression of *Shh* expression in the anterior domain [73]. In contrast, the hindlimb posterior domain is established by *Islet1* to regulate *Hand2* expression in this region [106].

Developmental analyses of limbless amphioxus and lampreys have provided clues to understand the evolutionary scenarios that led to the acquisition of limb-forming fields in the vertebrate body [4]. In amphioxus, the ventral mesoderm is not molecularly regionalized into cardiac versus posterior ventral mesoderm. In lamprey, the lateral plate mesoderm is regionalized into the ALPM and the PLPM, and *Hox* genes are expressed in a nested fashion in the PLPM [4], but the PLPM is not separated into the somatic and splanchnic layers [4]. These past and current findings suggest that, to acquire the limb-forming fields, novel expression domains of limb initiation genes in the lateral plate mesoderm, regionalization along its anterior–posterior axis, and its subdivision into somatic and splanchnic layers were likely to be required during vertebrate evolution.
